# Meat consumption is a major risk factor for hepatitis E virus infection

**DOI:** 10.1371/journal.pone.0176414

**Published:** 2017-04-27

**Authors:** Ed Slot, Hans L. Zaaijer, Michel Molier, Katja Van den Hurk, Femmeke Prinsze, Boris M. Hogema

**Affiliations:** 1 Sanquin Research, Department of Blood-borne infections, Amsterdam, the Netherlands; 2 Academic Medical Center, Department of Clinical Virology (CINIMA), Amsterdam, the Netherlands; 3 Sanquin Research, Department of Donor Studies, Amsterdam, the Netherlands; Centers for Disease Control and Prevention, UNITED STATES

## Abstract

**Background:**

The incidence of autochthonous hepatitis E virus genotype 3 (HEV gt3) infections in Western Europe is high. Although pigs are a major reservoir of the virus, the exact sources and transmission route(s) of HEV gt3 to humans remain unclear.

**Methods:**

To determine the role of meat consumption at a population level, the seroprevalence of anti-HEV IgG antibodies was compared between Dutch blood donors with a vegetarian lifestyle and donors who consume meat on a daily basis.

**Results:**

The age-weighted anti-HEV IgG seroprevalence among donors not eating meat was significantly lower than among meat-eating donors (12.4% vs 20.5%, p = 0.002). For both groups the prevalence strongly increased with age and the difference in prevalence was apparent for all age groups.

**Conclusions:**

Compared with meat-eating donors, the incidence of HEV infection is significantly lower among donors not eating meat, indicating that meat consumption is a major risk factor for HEV infection.

## Introduction

Hepatitis E virus (HEV) is an enterically transmitted non-enveloped virus, classified into at least 4 genotypes [1]. Recently it became clear that locally acquired HEV genotype 3 (gt3) infections occur frequently in high-income countries [1]. For example, in Southeast England, one in 2848 donations were found HEV RNA positive, and in the Netherlands one in 760 donations was HEV-RNA positive [2,3]. HEV gt3 infection usually goes unnoticed, but it can progress to severe and fatal disease; immunocompromised patients are at risk of developing chronic infection and cirrhosis [4]. Source(s) and transmission routes of HEV gt3 to humans are still enigmatic. Pigs form by far the largest known reservoir and are a likely source, as most swine farms throughout Europe are infected, HEV has been detected in retail meat and HEV isolates from pigs are genetically highly similar to HEV found in patients and blood donors [1,5]. However, the majority of sequences from HEV isolates from pigs in the United Kingdom differ from the dominant type seen in British patients, suggesting that HEV in the UK may be imported via meat from other countries, or additional sources and transmission routes may exist [6]. An increased seroprevalence among people with occupational contact with pigs (veterinarians and farmers) was reported in several but not all studies [7,8,9,10]. Case control studies have shown significantly more pork, wild boar and offal meat consumption among hepatitis E patients as compared to control groups [11,12], but part of the difference may be caused by recollection bias. Furthermore, these studies focused on patients displaying clinical symptoms, representing a minority of individuals infected with HEV gt3. Two local HEV outbreaks were studied in which asymptomatically infected persons were traced; consumption of pork liver pâté and shellfish were the most likely source of infection in a restaurant in Australia and on a cruise ship, respectively [13,14].

While consumption of undercooked meat is generally assumed to be a major source of HEV infection, most evidence is indirect or based on case reports. It is not well known to what extent HEV-contaminated fruit, vegetables and water contribute to transmission of HEV [15,16]. To further clarify the relation between meat consumption and HEV infection, we compared the anti-HEV IgG seroprevalence among blood donors who do not eat meat with those who eat meat on a daily basis.

## Materials and methods

Donors who consume meat on a daily basis and donors who declared to consume no meat at all were identified using data from Donor InSight, a questionnaire applied to a random sample of approximately 10% of the Dutch blood donor population in 2007–2009 [17]. As part of this questionnaire, donors were asked how often they consumed meat per week. Five answers were possible, ranging from ‘never’ to ‘(almost) every day’. 647 of 19,867 donors (3.3%) who answered the question about meat consumption declared to never consume meat and 18722 (94%) declared to consume meat ‘(almost) every day’. Repository samples from 403 donors who do now consume meat, collected between October 2011 and May 2012 were available. A control group with similar age and sex distribution was selected among donors who reported to consume meat on an (almost) daily basis (94.2% of the DIS participants) and made a donation between November 2012 and January 2013.

Samples were tested for HEV antibodies using the Wantai HEV-IgG assay (Wantai Biological Pharmacy Enterprise Co., Beijing, China) following the manufacturer’s instructions. Possible differences in sensitivity of different kit lots were assessed by testing a panel of samples representing the entire range of HEV-IgG antibody reactivity using the test kit lots applied. A comparison with the sensitivity of the kit lot used in our previous study [18] was made accordingly, using samples from the previous study and one of the kit lots used for the present study. Association between meat consumption and HEV prevalence was assessed by logistic regression analysis, adjusted for age and sex, using IBM SPSS statistics version 23 for Windows (IBM, Armonk, New York, USA).

All blood samples tested were collected from voluntary, non-remunerated repeat blood donors, who provided written informed consent as part of routine donor selection and blood collection procedures. The data on dietary lifestyle were collected from voluntary, non-remunerated repeat blood donors as part of the Donor InSight (DIS) cohort study, which was reviewed and approved by The Arnhem-Nijmegen Medical Ethical Committee and by the Ethical Advisory Council of Sanquin Blood Supply Foundation. All DIS participants gave their written informed consent.

## Results

**Fig 1:**
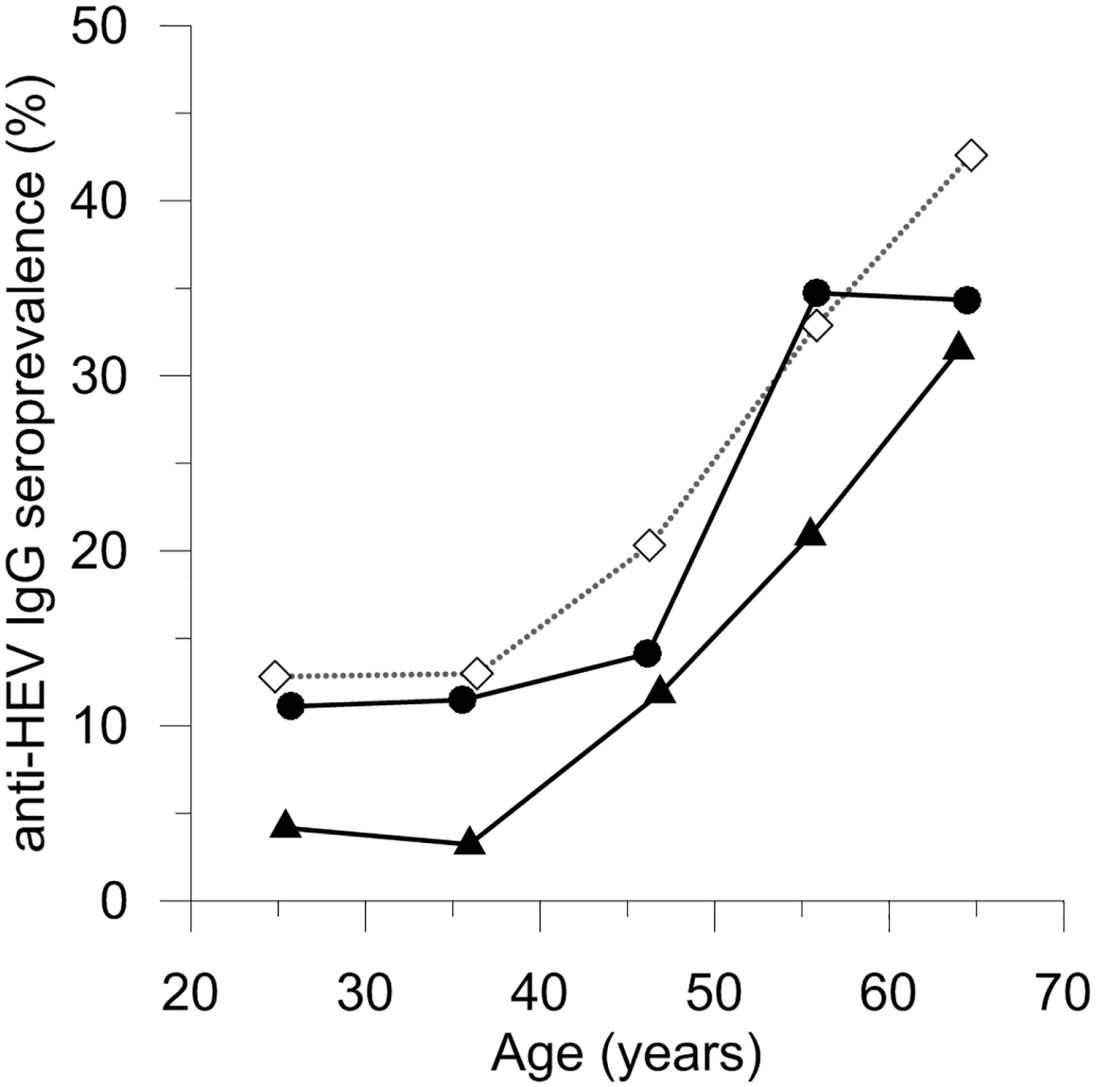
Anti-HEV prevalence in donors with a vegetarian lifestyle (triangles) and donors who consume meat on a daily basis (circles). For comparison, the age-dependent seroprevalence from our previous study is shown (diamonds) [18].

Fifty six of 403 (13.8%) plasma samples collected in 2011 and 2012 from Dutch blood donors who reported to consume no meat were anti-HEV IgG positive (Table 1, S1 Table), compared with 102/447 (22.8%) among the control group of donors who reported consuming meat on a daily basis (p = 0.001, Chi square). The age-weighted seroprevalence was 12.4% among vegetarian donors and 20.5% among meat-eating donors (age range 18–69 for both groups). No difference was observed between donors who consumed no meat and people who consume neither meat nor (shell)fish (data not shown). The anti-HEV seroprevalence strongly increases with age for both groups (Fig 1, Table 1). Logistic regression analysis showed that meat consumption was significantly associated with the presence of HEV antibodies (OR 1.78; 95%CI, 1.23–2.57; p = 0.002), independent of age and sex.

**Table 1:** Anti-HEV IgG prevalence among Dutch donors who do not eat meat and those who eat meat on a daily basis.

Age cohort	Meat eating donors			Non-meat eating donors		
	Male	Female	Total	Male	Female	Total
18–30	2/20 (10.0%)	8/70 (11.4%)	10/90 (11.1%)	1/18 (5.6%)	3/78 (3.8%)	4/96 (4.2%)
31–40	0/10 (0.0%)	7/51 (13.7%)	7/61 (11.5%)	1/12 (8.3%)	1/50 (2.0%)	2/62 (3.2%)
41–50	3/24 (12.5%)	9/61 (14.8%)	12/85 (14.1%)	3/20 (15%)	6/56 (10.7%)	9/76 (11.8%)
51–60	21/62 (33.9%)	29/82 (35.4%)	50/144 (34.7%)	8/48 (16.7%)	16/67 (23.9%)	24/115 (20.9%)
61–70	11/27 (40.7%)	12/40 (30.0%)	23/67 (34.3%)	7/24 (29.2%)	10/30 (33.3%)	17/54 (31.5%)
Total	37/143 (25.9%)	65/304 (21.4%)	102/447 (22.8%)	20/122 (16.4%)	36/281 (12.8%)	56/403 (13.9%)

Three different Wantai HEV IgG kit lots (designated A-C) were used for this study. The sensitivity of the lots was compared by measuring panels of 11–15 samples with varying reactivity ranging from the cut-off to maximum signal, using the different lots. Data were compared by linear regression. With one exception the correlation between the measurements was good, with linear regression slopes between 0.98 and 1.02 and correlation coefficients >0.94. However, the reactivity with one lot (lot C) was consistently ~35% lower (slope 0.64, correlation coefficient 0.99). All 363 samples measured with the deviant lot were re-tested using lot A, resulting in 12 discrepant results (samples initially testing negative were in fact weakly positive).

## Discussion

Our study shows that the anti-HEV IgG seroprevalence is significantly lower among donors who do not eat meat, versus those who do on a daily basis. Although pigs have long been recognized as the largest known reservoir for HEV gt3, little is known about the route(s) of infection to humans, since most human cases of clinical HEV infection are not directly associated with pigs or pig products. The long period between infection and appearance of clinical symptoms makes it difficult to find the sources of infection. Various case control studies showed significantly more consumption of pork, wild boar and offal meat consumption among hepatitis E patients compared to control groups, and even a British supermarket chain could be identified as a potential source of gt3 HEV infection [11,12]. Identification of shellfish as the cause of a large outbreak on a cruise ship and presence of HEV on berries provide evidence that meat is not the only source of infection [14,16].

To our knowledge the seroprevalence among vegetarians has not been used previously to assess the role of meat consumption in HEV infection. We observed a 42% lower prevalence of past HEV infection in vegetarian blood donors compared to donors consuming meat. Our study is the first to provide a minimum estimate of the extent to which meat consumption is associated with HEV infection among healthy people using a non-retrospective approach.

The seroprevalence in meat-eating donors was very similar to that observed previously among randomly selected donors in our anti-HEV seroprevalence study conducted in 2011 [18]. In this study 1401 of 5239 donors (26.7%) were HEV IgG positive, and the age-weighted seroprevalence was 24.0%. Considering the large proportion of donors who consume meat on a daily basis it is not surprising that the low percentage (3.3%) of vegetarian donors is not sufficient to affect the overall seroprevalence. Surprisingly, the difference in seroprevalence was similar among all age groups, suggesting that vegetarians may be protected against the recent increase in HEV incidence in the Netherlands that occurred after a long period of lower HEV incidence [19].

We excluded the possibility that differences between the groups were caused by differences in sensitivity of the different ELISA lots. One lot showed significantly reduced sensitivity though, and all samples were re-tested. The observation that differences between lots exist suggest that results from different studies cannot always be compared directly. However, because only a limited proportion of samples displays reactivity around the cutoff the effect of differences in sensitivity are expected to be limited. Only 5.3% of donors in this study show reactivity between 0.5 and 1.5 times the cut-off. We compared the sensitivity of the kit lots using panels of samples to compare results with our previous study. Using the WHO standard may be a more convenient method; we confirmed the results from Abravanel and colleagues who reported that the cut-off of the Wantai test is approximately 0.25 IU/mL [20].

We emphasize that the difference in HEV prevalence between vegetarian donors and donors who consume meat on a daily basis was observed despite a very basic assessment of the vegetarianism, not including questions about the vegetarian lifestyle history. In a Dutch cohort study approximately 50% of self-reported vegetarians had adhered to this lifestyle for less than ten years [21]. Assuming that the same applies to the vegetarians in the Dutch blood donor population, it seems likely that the vegetarian blood donors consumed meat for a significant part of their life. The effect of meat consumption on anti HEV prevalence may therefore be underestimated, in particular among older donors.

We conclude that eating meat is highly associated with hepatitis E virus infection in the Netherlands. This may also apply to other industrialized countries. However, the identification of meat as a major risk factor for HEV infection does not exclude a role of other transmission routes (e.g. via crops or fruit that have been in contact with pig manure or HEV-contaminated water) [16]. More detailed studies are required to address the source(s) of HEV in more detail. Meanwhile, the outcome of our study stresses the importance of safe meat production and preparation procedures to ensure that undercooking and cross-contamination to other foods are prevented. It is urgently needed to bring this under the attention of both the food industry and the general public. Finally, measures to reduce circulation of this human pathogen in commercial pig farms should be considered.

## Supporting information

S1 Table Primary data of the anti-HEV test at participant level. (XLSX)
